# The Effect of 1,8-Cineole Inhalation on Preoperative Anxiety: A Randomized Clinical Trial

**DOI:** 10.1155/2014/820126

**Published:** 2014-06-16

**Authors:** Ka Young Kim, Hyo Jin Seo, Sun Seek Min, Mira Park, Geun Hee Seol

**Affiliations:** ^1^Department of Basic Nursing Science, School of Nursing, Korea University, 145 Anam-ro, Seongbuk-gu, Seoul 136-701, Republic of Korea; ^2^Department of Physiology and Biophysics, School of Medicine, Eulji University, Daejeon 301-746, Republic of Korea; ^3^Department of Premedicine, School of Medicine, Eulji University, Daejeon 301-746, Republic of Korea

## Abstract

The aim of this study was to investigate the effect of inhalation of eucalyptus oil and its constituents on anxiety in patients before selective nerve root block (SNRB). This study was a randomized controlled trial carried out in 62 patients before SNRB. The patients were randomized to inhale limonene, 1,8-cineole, or eucalyptus oil, each at concentrations of 1% vol/vol in almond oil or almond oil (control). Anxiety-visual analog scale (A-VAS), state-trait anxiety inventory (STAI), profile of mood states (POMS), pain-visual analog scale (P-VAS), blood pressure, and pulse rate were measured before and after inhalation prior to SNRB. Measures of anxiety, including A-VAS (*P* < 0.001), STAI (*P* = 0.005), and POMS (*P* < 0.001), were significantly lower in 1,8-cineole than in the control group and significantly greater in 1,8-cineole than in the eucalyptus group in A-VAS. P-VAS was significantly lower after than before inhalation of limonene, 1,8-cineole, and eucalyptus, despite having no significant difference in the four groups compared with control group. 1,8-Cineole, a major constituent of eucalyptus, was effective in decreasing anxiety before SNRB. The present findings suggest that inhalation of 1,8-cineole may be used to relieve anxiety before, during, and after various operations, in addition to SNRB.

## 1. Introduction

Anxiety is common in patients prior to surgery, with high anxiety levels negatively influencing postoperative outcomes. For example, high preoperative anxiety after spinal anesthesia in women undergoing cesarean delivery has been associated with a greater reduction in systolic arterial pressure [1]. In addition, preoperative anxiety in children has been found to be related to increased postoperative pain [2].

Anxiety is modulated by pharmacological approaches [3]. Preoperative administration of melatonin has been shown to be effective in reducing this anxiety [4]. However, since local anesthesia, unlike general anesthesia, cannot completely control anxiety during surgery, it is essential to manage preoperative anxiety appropriately in patients undergoing operations, such as selective nerve root block (SNRB), under local anesthesia.

Aromatherapy is a type of complementary and alternative medicine, used broadly in the management of chronic pain, depression, anxiety, insomnia, and stress-related disorders [5, 6]. Essential oils are absorbed into the olfactory and respiratory systems via inhalation or into the transcutaneous system via massage and bathing [7]. Inhalation of essential oils transmits signals from the olfactory system to the brain, which regulates anxiety, depression, and mood disorders by secreting neurotransmitters such as serotonin and dopamine [5]. Despite its use in pain relief, psychological comfort, and disease prevention, evidence for the therapeutic efficacy of aromatherapy remains poor [6].

Eucalyptus oils used in aromatherapy have antioxidant, anti-inflammatory, and antimicrobial properties [8]. The components of eucalyptus oil include 1,8-cineole (61.46%), limonene (13.68%), *ρ*-cymene (8.55%), *γ*-terpinene (5.87%), *α*-pinene (4.95%), and *α*-phellandrene (1.09%) [9]. Moreover, ellagic acid, a polyphenolic component of eucalyptus oil, has been reported to produce an antidepressant-like effect [10], as well as anxiolytic activity with inhibition of the depressant action [11].

Essential oils and their constituents have been used for medicinal and pharmaceutical effects. 1,8-Cineole, the major active constituent of eucalyptus, is a small lipophilic molecule that easily passes across the blood-brain barrier and may show effects at the neuronal level by acting on receptor sites and enzyme activity [12]. This compound showed stimulant activity in mice, significantly increasing ambulatory activity [13] and reflecting a reduced level of anxiety [14]. Furthermore, 1,8-cineole was reported to inhibit the activity of acetylcholinesterase (AChE) [15], a nervous system enzyme that catalyzes the hydrolysis of the neurotransmitter acetylcholine to transmit nerve impulses [16]. Since the cholinergic system has been associated with anxiety [17], we assessed whether eucalyptus and its major constituents have an effect on anxiety before SNRB.

## 2. Materials and Methods

### 2.1. Study Design and Sample Size

**Figure 1:**
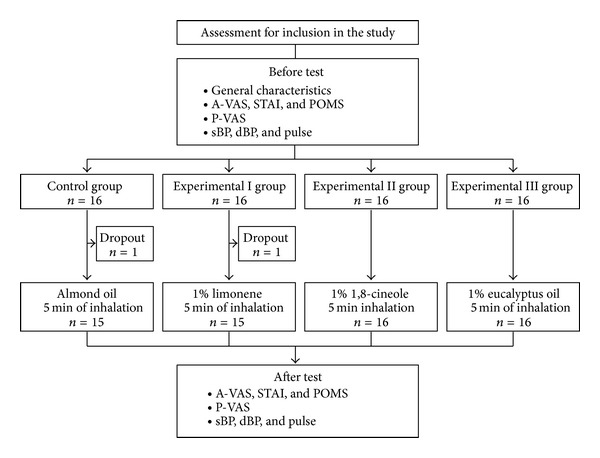
Research design.

In this randomized, controlled trial (Figure 1), patients were randomized to inhale 1% (v/v, dissolved in almond oil) limonene, 1% (v/v) 1,8-cineole, 1% (v/v) eucalyptus oil, or almond oil (control) for 5 minutes before SNRB. Patients and investigators were not informed about the types and effects of aroma oil. Patients were randomly assigned to an experimental or control group using a table of random numbers. Based on an effect size of 0.40, a statistical power of 0.70, and a significance level of 0.05, we calculated that the minimum sample size needed to compare differences among four groups was >15 subjects per group. Sixty-four subjects were originally assigned to each group; however, 1 patient in the control group and 1 in the 1% limonene group dropped out. Two patients who ceased taking inhalation for personal reasons were excluded. Thus, data were collected from 62 patients.

### 2.2. Participants

The study was approved by the Research Ethics Review Committee of Korea University Medical Center (Code: ED12172). Participants who met the inclusion criteria and provided written informed consent were enrolled. All subjects were conscious and oriented, were not being treated with any anxiolytic or antidepressant agent, and had not been prescribed hormonotherapy or aromatherapy. In addition, none had asthma or an allergic reaction to any of the aromas used in this study or any trouble with sense of smell.

### 2.3. Intervention

Almond oil (Aromarant Co. Ltd., Rottingen, Germany), 5% eucalyptus oil (Aromarant Co. Ltd., Rottingen, Germany), 1% limonene (Sigma Aldrich, Steinheim, Germany), and 1% 1,8-cineole (Sigma Aldrich, Steinheim, Germany) were prepared. One mL aliquot of each was dropped onto aroma pads, which were positioned 30 cm from the tip of the nose. Patients were instructed to inhale comfortably and naturally for 5 minutes, complete all the tests outlined below, and then undergo SNRB 20 minutes later. All experiments were carried out separately. The compounder was the only one who knows which participant affiliated to which group according to the assigned number on bottle. Patients and investigator were not informed about the types and effects of aroma oil.

### 2.4. Anxiety-Visual Analog Scale (A-VAS), State-Trait Anxiety Inventory (STAI), Profile of Mood States (POMS), and Pain-Visual Analog Scale (P-VAS)

Visual analog scales (VAS) have been used in psychological assessments and in a wide variety of health-related constructs, including pain, quality of life, and mood. Preoperative anxiety was measured using the anxiety-visual analog scale (A-VAS), a horizontal scale, ranging from 0 (no pain) on the left side to 10 (extreme pain) on the right side. Patients were asked to indicate an A-VAS score by number at each determination. The state-trait anxiety inventory (STAI) is a self-reported questionnaire consisting of two 20-item scales used to determine the levels of state anxiety and trait anxiety. Each item was scored from one to four points (not at all, somewhat, moderate, and very much), with higher scores indicating greater anxiety. Profile of mood states (POMS) is a self-reported questionnaire assessing mood states and is categorized into six scales (anger hostility, tension anxiety, depression dejection, vigor activity, fatigue inertia, and confusion bewilderment) and a total mood disturbance score. Each item was scored using a 5-point Likert scale ranging from 0 (not at all) to 4 (extremely).

Pain-VAS consists of a horizontal scale, ranging from 0 (no pain) on the left side to 10 (extreme pain) on the right side. Patients were asked to indicate a P-VAS score by number at each determination. These methods are suitable and reliable for patients who can describe their anxiety, mood, and pain conditions [18].

### 2.5. Measurement of Blood Pressure and Pulse Rate

Blood pressure and pulse rate were measured before and after aroma inhalation, as indicators of the reaction of the autonomic nervous system to anxiety and pain. Blood pressure was measured in the brachial artery using an electronic sphygmomanometer (model BP 3BMI-3, Microlife, Switzerland) after a 20-minute rest in a supine position. Pulse was measured at the radial artery for 1 minute.

### 2.6. Statistical Analysis

SPSS 20.0 was used for statistical analyses. Categorical and continuous variables were compared using Fisher's exact test and chi-square test, respectively. Differences among groups were assessed by one-way analysis of variance (ANOVA) and the Kruskal-Wallis test, and differences within each group were compared by Wilcoxon and paired *t*-tests. A *P* value <0.05 was defined as statistically significant.

## 3. Results

### 3.1. General Characteristics of Subjects and the Homogeneity Test

The baseline characteristics of the four groups are shown in Table 1. The mean age of the subjects was 53.7 years. There were 25 men (40.3%) and 37 women (59.7%) in all patients. Of the 62 subjects, 51 patients (82.3%) were married. Regarding medication, 32 subjects (51.6%), 7 subjects (11.3%), and 26 subjects (51.9%) took analgesic, antihypertensive drug, and antidiabetic drug, respectively. Seventeen patients (27.4%) had procedure history. Overall, there were no significant differences in demographic characteristics, procedure history, or medications, including analgesic, antihypertensive, and antidiabetic drugs. Moreover, prior to inhalation, there were no significant differences among the four groups in scores on the A-VAS, STAI, POMS, and P-VAS or differences in systolic blood pressure (sBP), diastolic blood pressure (dBP), or pulse (Table 2).

### 3.2. Effect of Inhalation on A-VAS

**Figure 2:**
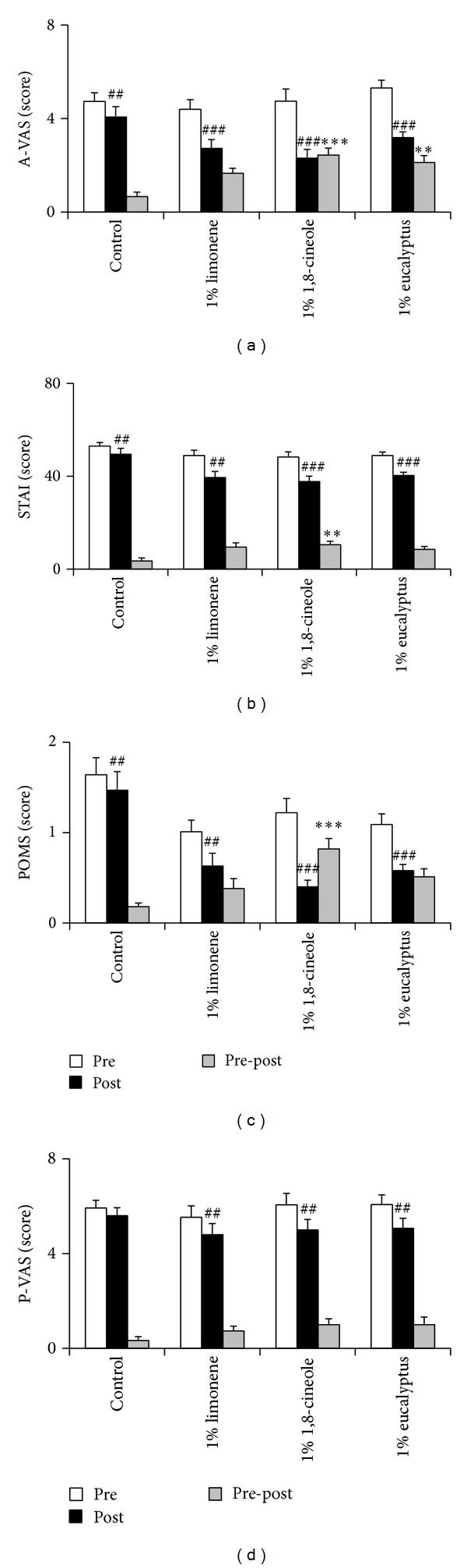
Effect of limonene, 1,8-cineole, or eucalyptus inhalation on (a) anxiety-visual analog scale (A-VAS), (b) state-trait anxiety inventory (STAI), (c) profile of mood states (POMS), and (d) pain-visual analog scale (P-VAS). Data represent mean values ± SEM (*n* = 15-16 per group). ^##^ *P* < 0.01, ^###^ *P* < 0.001 compared with subjects in the same group before inhalation; **P < 0.01, ***P < 0.001 compared with the control group.

A-VAS was significantly lower after than before inhalation of almond (4.07 ± 0.44 versus 4.73 ± 0.37 points, *P* = 0.008), limonene (2.73 ± 0.37 versus 4.40 ± 0.41 points, *P* < 0.001), 1,8-cineole (2.31 ± 0.37 versus 4.75 ± 0.51 points, *P* < 0.001), and eucalyptus (3.19 ± 0.25 versus 5.31 ± 0.33, *P* < 0.001, Figure 2(a)). The decreases were significantly greater for 1,8-cineole (2.44 ± 0.30 points, *P* < 0.001) and eucalyptus (2.13 ± 0.29 points, *P* = 0.001) than for almond (0.67 ± 0.19 points) and were significantly greater for 1,8-cineole than for eucalyptus (Figure 2(a)).

### 3.3. Effect of Inhalation on STAI and POMS

STAI was significantly lower after than before inhalation of almond (49.47 ± 2.55 versus 53.00 ± 1.55 points, *P* = 0.006), limonene (39.47 ± 2.58 versus 48.93 ± 2.25 points, *P* = 0.001), 1,8-cineole (37.75 ± 2.30 versus 48.25 ± 2.26 points, *P* < 0.001), and eucalyptus (40.31 ± 1.42 versus 48.94 ± 1.54 points, *P* < 0.001, Figure 2(b)). POMS was significantly lower after than before inhalation of almond (1.47 ± 0.21 versus 1.64 ± 0.19 points, *P* = 0.001), limonene (0.63 ± 0.14 versus 1.01 ± 0.13 points, *P* = 0.002), 1,8-cineole (0.40 ± 0.07 versus 1.22 ± 0.16 points, *P* < 0.001), and eucalyptus (0.58 ± 0.07 versus 1.09 ± 0.12 points, *P* < 0.001, Figure 2(c)). Moreover, 1,8-cineole showed significantly greater decreases than control on the STAI (10.50 ± 1.55 versus 3.53 ± 1.26 points, *P* = 0.005, Figure 2(b)) and POMS (0.82 ± 0.12 versus 0.18 ± 0.04 points, *P* < 0.001, Figure 2(c)).

### 3.4. Effect of Inhalation on P-VAS

P-VAS scores were significantly lower after than before inhalation of 1% limonene (4.80 ± 0.47 versus 5.53 ± 0.49 points, *P* = 0.008), 1% 1,8-cineole (5.00 ± 0.44 versus 6.06 ± 0.48 points, *P* = 0.002), and 1% eucalyptus (5.07 ± 0.42 versus 6.07 ± 0.42 points, *P* = 0.008, Figure 2(d)), but not almond oil, with none of the between-group differences being statistically significant (*P* = 0.125, Figure 2(d)).

### 3.5. Effects of Inhalation on Blood Pressure and Pulse

sBP was significantly lower after than before inhalation of 1,8-cineole (*P* = 0.032) and eucalyptus (*P* = 0.012), but none of the between-group differences was statistically significant (Table 3). Inhalation, however, had no effect on dBP or pulse.

## 4. Discussion

This study focused on the anxiolytic effects of eucalyptus and its constituents, limonene, and 1,8-cineole using three different tools, the A-VAS, STAI, and POMS scales, in subjects scheduled for SNRB. In addition, the study assessed pain score, blood pressure, and pulse. Inhalation of eucalyptus, limonene, 1,8-cineole, or almond oil significantly decreased preoperative anxiety on all three scales; however, the decreases following the inhalation of 1,8-cineole were greater than those of the control group. Moreover, the decrease on the A-VAS was greater following the inhalation of 1,8-cineole than of eucalyptus oil.

1,8-Cineole, a monoterpene, has been reported to have several biological effects including an anxiolytic effect. Moreover, 1,8-cineole has been reported to be a hypotensive agent and smooth muscle relaxant as well as a modulator of neural firing in areas of the olfactory lobe [19, 20]. 1,4-Cineole, a natural monoterpene analog of 1,8-cineole, showed an anxiolytic-like effect in the elevated plus maze, hole board, and open-field tests [21]. Inhalation of* Alpinia zerumbet* essential oil, which contains 1,8-cineole, had anxiolytic effects in mice [22]. The essential oils of eucalyptus were found to decrease the avoidance rate in mice without affecting their response on a discrete shuttle-type task, a type of conditioned avoidance task [23]. In agreement with previous findings in mice, our results indicate that 1,8-cineole may exert anxiolytic effects in humans.

We found that anxiety was decreased in our control group, perhaps due to emotional support and comfort by researchers and nurses. However, the decrease in anxiety was significantly greater in patients inhaling 1,8-cineole than in the control group, suggesting that inhalation of this compound may be effective in managing preoperative anxiety in patients undergoing surgery, such as SNRB, under local anesthesia.

Administration of 1,8-cineole 400 mg/kg effectively blocked response in both early and late phases in a manner similar to morphine in formalin tests, a model of clinical pain [24]. In addition, 1,8-cineole is a natural antagonist of human transient receptor potential A1 (TRPA1), which functions as a receptor for noxious cold temperatures and somatic mechanosensations, with 1,8-cineole having analgesic effects by inhibiting TRPA1 [25]. Eucalyptus oil, which contains both limonene and 1,8-cineole, was found to have antinociceptive effects on acetic acid-induced writhing and hot-plate induced thermal simulation tests in mice [8]. Furthermore, inhalation of 3% eucalyptus oil every 30 minutes for 3 days after total knee replacement effectively reduced pain and inflammatory responses [9].

In contrast, we found that although pain was reduced in each of the four groups there were no significant between-group differences. Stress-induced analgesia (SIA) has been found to suppress pain in patients exposed to unconditioned or conditioned stressful stimuli, an effect mediated by the activation of the descending inhibitory pain pathway. Patients experiencing high stress experience less pain, due to stress-induced analgesia, a process associated with the release of the endogenous opioid *β*-endorphin, a natural pain reliever. Preoperative anxiety was found to increase the plasma concentration of *β*-endorphin [26], with SIA having an antinociceptive effect. The reduction in nociceptive activity may be characterized by a relative increase in pain perception due to a reduction in anxiety after inhalation of 1,8-cineole or eucalyptus. However, inhalation of eucalyptus oil for 5 minutes before surgery may be insufficient to exert antinociceptive effects [9].

Preoperative anxiety generally stimulates sympathetic systems, leading to increases in blood pressure and heart rate. We found that inhalation of 1,8-cineole and eucalyptus significantly decreased SBP due to the anxiolytic effect of 1,8-cineole, but the differences between groups were not significant. 1,8-Cineole administration has also been shown to reduce gastric compliance, arterial pressure, and cardiac frequency in anesthetized rats [27]. Furthermore, a high dose (10 mg/kg) of 1,8-cineole was associated with hypotension and bradycardia in both anesthetized and conscious rats [20]. Eucalyptus oils showed antihypertensive and vascular relaxation effects on rat aortae [28], as well as reducing blood pressure and heart rate [9]. However, we found that the antinociceptive effects of 1,8-cineole and eucalyptus did not differ significantly before SNRB, with both reducing SIA.

This study had several limitations. First, patients were randomly allocated to study groups and test oils were coded by an individual independent of this study. Although patients and treating investigators were not informed about the types and effects of aroma oils, patients may have recognized the smell of the test oils to which they were randomized, which may have affected their responses. Second, the sample size of this study was relatively small. Third, the use of a randomized controlled trial in this context may raise ethical issues, limiting the results.

In conclusion, we have shown that inhalation of 1,8-cineole, a major constituent of eucalyptus, was effective in reducing preoperative anxiety and pain in patients prior to SNRB. These findings suggest that inhalation of 1,8-cineole or eucalyptus may be utilized therapeutically to relieve anxiety before, during, and after various operations, including SNRB.

## Figures and Tables

**Table 1:** Demographic and general characteristics of the subjects *N* = 62.

Characteristics	Total *N* (%)	Control *n* (%)	Limonene *n* (%)	1,8-Cineole *n* (%)	Eucalyptus *n* (%)	*χ* ^2^	*P* value
Age (year)							
30–39	9 (14.5)	0 (0.0)	4 (44.4)	2 (22.2)	3 (33.3)	7.81	0.568^a^
40–49	16 (25.8)	3 (18.75)	3 (18.75)	4 (25.00)	6 (37.50)		
50–59	11 (17.7)	3 (27.27)	2 (18.18)	4 (36.36)	2 (18.18)		
≥60	26 (41.9)	9 (34.62)	6 (23.08)	6 (23.08)	5 (19.23)		
Gender							
Male	25 (40.3)	5 (20.00)	6 (24.00)	8 (32.00)	6 (24.00)	0.98	0.806
Female	37 (59.7)	10 (27.03)	9 (24.32)	8 (21.62)	10 (27.03)		
Education							
≤middle	16 (25.8)	6 (37.50)	4 (25.00)	5 (31.25)	1 (6.25)	7.85	0.225^a^
High school	21 (33.9)	4 (19.05)	3 (14.29)	7 (33.33)	7 (33.33)		
≥college	25 (40.3)	5 (20.00)	8 (32.00)	4 (16.00)	8 (32.00)		
Religion							
Christian	19 (30.7)	5 (26.32)	4 (21.05)	4 (21.05)	6 (31.58)	5.79	0.788^a^
Catholic	11 (17.7)	1 (9.09)	4 (36.36)	2 (18.18)	4 (36.36)		
Buddhist	14 (22.6)	5 (35.71)	2 (14.29)	5 (35.71)	2 (14.29)		
None	18 (29.0)	4 (22.22)	5 (27.78)	5 (27.78)	4 (22.22)		
Marital state							
Married	51 (82.3)	14 (27.45)	11 (21.57)	14 (27.45)	12 (23.53)	2.96	0.440^a^
Not married	11 (17.7)	1 (9.09)	4 (36.36)	2 (18.18)	4 (36.36)		
Analgesic							
Yes	32 (51.6)	6 (18.75)	6 (18.75)	9 (28.13)	11 (34.40)	3.64	0.303^a^
No	30 (48.4)	9 (30)	9 (30)	7 (23.33)	5 (16.67)		
Antihypertensive drug							
Yes	7 (11.3)	2 (28.57)	3 (42.86)	2 (28.57)	0 (0.0)	3.26	0.353
No	55 (88.7)	13 (23.64)	12 (21.82)	14 (25.45)	16 (29.09)		
Antidiabetic drug							
Yes	26 (51.9)	6 (23.08)	9 (34.62)	5 (19.23)	6 (23.08)	2.91	0.405^a^
No	36 (58.1)	9 (25.00)	6 (16.67)	11 (30.56)	10 (27.78)		
Procedure history							
Yes	17 (27.4)	4 (23.53)	5 (29.41)	4 (23.53)	4 (23.53)	0.36	0.960
No	45 (72.6)	11 (24.44)	10 (22.22)	12 (26.67)	12 (26.67)		

Analyzed using ^a^Fisher's exact test and chi-square test.

*n* = 15 each in the control and limonene groups and 16 each in the 1,8-cineole and eucalyptus groups.

**Table 2:** Homogeneity test for measurement variables among the four groups *N* = 62.

Variable	Control	Limonene	1,8-Cineole	Eucalyptus	*P* value
A-VAS	4.73 ± 1.44	4.40 ± 1.59	4.75 ± 2.05	5.31 ± 1.30	0.466^a^
STAI	53.00 ± 6.01	48.93 ± 8.72	48.25 ± 9.05	48.94 ± 6.14	0.305
POMS	1.64 ± 0.73	1.01 ± 0.59	1.22 ± 0.64	1.09 ± 0.47	0.070^a^
P-VAS	5.93 ± 1.22	5.53 ± 1.88	6.06 ± 1.88	6.19 ± 1.68	0.617^a^
sBP	129.40 ± 11.16	125.67 ± 7.58	136.00 ± 11.72	134.81 ± 16.38	0.076
dBP	84.47 ± 11.26	82.93 ± 7.47	85.50 ± 10.27	85.31 ± 15.32	0.923
Pulse	79.33 ± 6.89	76.13 ± 6.71	78.81 ± 10.39	79.13 ± 6.87	0.657

Analyzed using one-way ANOVA and Kruskal-Wallis test^a^.

Data presented as mean ± SD.

*n* = 15 each in the control and limonene groups and 16 each in the 1,8-cineole and eucalyptus groups.

**Table 3:** Effects of inhalation of limonene, 1,8-cineole, or eucalyptus on systolic blood pressure (sBP), diastolic blood pressure (dBP), and pulse *N* = 62.

Variables	Control	Limonene	1,8-Cineole	Eucalyptus	*P* value
sBP (mmHg)					
Pre	129.4 ± 11.16	125.67 ± 7.58	136.00 ± 11.72	134.81 ± 16.38	0.173
Post	129.13 ± 11.69	124.40 ± 13.29	131.63 ± 11.87	130.13 ± 12.71	
Difference	0.27 ± 4.06	1.27 ± 7.89	4.38 ± 7.40	4.69 ± 6.52	
*P* value	0.803	0.544	0.032	0.012	
dBP (mmHg)					
Pre	84.47 ± 11.26	82.93 ± 7.47	85.50 ± 10.27	85.31 ± 15.32	0.229
Post	84.00 ± 11.99	80.33 ± 8.89	87.19 ± 8.32	85.50 ± 12.23	
Difference	0.47 ± 3.09	2.60 ± 4.88	−1.69 ± 7.57	−0.19 ± 6.24	
*P* value	0.568	0.058	0.387	0.906	
Pulse (beats/min)					
Pre	79.33 ± 6.89	76.13 ± 6.71	78.81 ± 10.39	79.13 ± 6.87	0.179
Post	77.13 ± 5.00	75.33 ± 7.27	79.94 ± 11.53	78.56 ± 6.88	
Difference	2.20 ± 5.19	0.80 ± 2.60	−1.13 ± 3.63	0.56 ± 4.63	
*P* value	0.123	0.253	0.234	0.634	

Pre: before inhalation; post: after inhalation.

Data presented as mean ± SD.

*n* = 15 each in the control and limonene groups and 16 each in the 1,8-cineole and eucalyptus groups.

## References

[1] Orbach-Zinger S, Ginosar Y, Elliston J (2012). Influence of preoperative anxiety on hypotension after spinal anaesthesia in women undergoing Caesarean delivery. *British Journal of Anaesthesia*.

[2] Kain ZN, Mayes LC, Caldwell-Andrews AA, Karas DE, McClain BC (2006). Preoperative anxiety, postoperative pain, and behavioral recovery in young children undergoing surgery. *Pediatrics*.

[3] Pritchard M (2010). Measuring anxiety in surgical patients using a visual analogue scale. *Nursing Standard*.

[4] Maitra S, Baidya DK, Khanna P (2013). Melatonin in perioperative medicine: current perspective. *Saudi Journal of Anaesthesia*.

[5] Lv XN, Liu ZJ, Zhang HJ, Tzeng CM (2013). Aromatherapy and the central nerve system (CNS): therapeutic mechanism and its associated genes. *Current Drug Targets*.

[6] Zhang Y, Wu Y, Chen T (2013). Assessing the metabolic effects of aromatherapy in human volunteers. *Evidence-Based Complementary and Alternative Medicine*.

[7] Cavanagh HMA, Wilkinson JM (2002). Biological activities of lavender essential oil. *Phytotherapy Research*.

[8] Atta AH, Alkofahi A (1998). Anti-nociceptive and anti-inflammatory effects of some Jordanian medicinal plant extracts. *Journal of Ethnopharmacology*.

[9] Jun YS, Kang P, Min SS, Lee J-M, Kim H-K, Seol GH (2013). Effect of eucalyptus oil inhalation on pain and inflammatory responses after total knee replacement: a randomized clinical trial. *Evidence-Based Complementary and Alternative Medicine*.

[10] Girish C, Raj V, Arya J, Balakrishnan S (2012). Evidence for the involvement of the monoaminergic system, but not the opioid system in the antidepressant-like activity of ellagic acid in mice. *European Journal of Pharmacology*.

[11] Quílez AM, Saenz MT, García Giménez MD (2012). Uncaria tomentosa (Willd. ex. Roem. & Schult.) DC. and Eucalyptus globulus Labill. interactions when administered with diazepam. *Phytotherapy Research*.

[12] Moss M, Oliver L (2012). Plasma 1,8-cineole correlates with cognitive performance following exposure to rosemary essential oil aroma. *Therapeutic Advances in Psychopharmacology*.

[13] Umezu T, Sakata A, Ito H (2001). Ambulation-promoting effect of peppermint oil and identification of its active constituents. *Pharmacology Biochemistry and Behavior*.

[14] Lau AA, Crawley AC, Hopwood JJ, Hemsley KM (2008). Open field locomotor activity and anxiety-related behaviors in mucopolysaccharidosis type IIIA mice. *Behavioural Brain Research*.

[15] Savelev S, Okello E, Perry NSL, Wilkins RM, Perry EK (2003). Synergistic and antagonistic interactions of anticholinesterase terpenoids in Salvia lavandulaefolia essential oil. *Pharmacology Biochemistry and Behavior*.

[16] Lionetto MG, Caricato R, Calisi A, Giordano ME, Schettino T (2013). Acetylcholinesterase as a biomarker in environmental and occupational medicine: New insights and future perspectives. *BioMed Research International*.

[17] File SE, Gonzalez LE, Andrews N (1998). Endogenous acetylcholine in the dorsal hippocampus reduces anxiety through actions on nicotinic and muscarinic receptors. *Behavioral Neuroscience*.

[18] Rossi V, Pourtois G (2012). Transient state-dependent fluctuations in anxiety measured using STAI, POMS, PANAS or VAS: a comparative review. *Anxiety, Stress and Coping*.

[19] Nikitin ES, Balaban PM (2000). Optical recording of odor-evoked responses in the olfactory brain of the naive and aversively trained terrestrial snails. *Learning and Memory*.

[20] Lahlou S, Figueiredo AF, Magalhães PJC, Leal-Cardoso JH (2002). Cardiovascular effects of 1,8-cineole, a terpenoid oxide present in many plant essential oils, in normotensive rats. *Canadian Journal of Physiology and Pharmacology*.

[21] Gomes PB, Feitosa ML, Silva MIG (2010). Anxiolytic-like effect of the monoterpene 1,4-cineole in mice. *Pharmacology Biochemistry and Behavior*.

[22] de Araújo FYR, Silva MIG, Moura BA (2009). Central nervous system effects of the essential oil of the leaves of Alpinia zerumbet in mice. *Journal of Pharmacy and Pharmacology*.

[23] Umezu T (2012). Evaluation of the effects of plant-derived essential oils on central nervous system function using discrete shuttle-type conditioned avoidance response in mice. *Phytotherapy Research*.

[24] Santos FA, Rao VS (2000). Antiinflammatory and antinociceptive effects of 1, 8-cineole a terpenoid oxid present in many plant essential oils. *Phytotherapy Research*.

[25] Takaishi M, Fujita F, Uchida K (2012). 1,8-cineole, a TRPM8 agonist, is a novel natural antagonist of human TRPA1. *Molecular Pain*.

[26] Butler RK, Finn DP (2009). Stress-induced analgesia. *Progress in Neurobiology*.

[27] Neves JRC, de Lira GHS, Neto RMDO (2007). 1.8 Cineole decreases gastric compliance in anesthetized rats. *Acta Cirurgica Brasileira*.

[28] Yvon Y, Guy Raoelison E, Razafindrazaka R (2012). Relation between chemical composition or antioxidant activity and antihypertensive activity for six essential oils. *Journal of Food Science*.

